# *N*-Acetylcysteine improves intestinal function and attenuates intestinal autophagy in piglets challenged with β-conglycinin

**DOI:** 10.1038/s41598-021-80994-2

**Published:** 2021-01-13

**Authors:** Huiyun Wang, Chengcheng Li, Meng Peng, Lei Wang, Di Zhao, Tao Wu, Dan Yi, Yongqing Hou, Guoyao Wu

**Affiliations:** 1 Hubei International Scientific and Technological Cooperation Base of Animal Nutrition and Gut Health, Wuhan Polytechnic University, Wuhan, 430023 China; 2 Department of Animal Science, Texas A&M University, College Station, TX 77843 USA

**Keywords:** Animal physiology, Jejunum

## Abstract

β-Conglycinin (β-CG), an anti-nutritional factor, is a major allergen in soybeans to induce intestinal dysfunction and diarrhea in neonatal animals, including piglets and human infants. This study with a piglet model determined the effects of *N*-acetylcysteine (NAC) on intestinal function and autophagy in response to β-CG challenge. Twenty-four 12-day-old piglets (3.44 ± 0.28 kg), which had been weaned at 7 days of age and adapted for 5 days after weaning, were randomly allocated to the control, β-CG, and β-CG + NAC groups. Piglets in the control group were fed a liquid diet containing 10% casein, whereas those in the β-CG and β-CG + NAC groups were fed the basal liquid diets containing 9.5% casein and 0.5% β-CG for 2 days. Thereafter, pigs in the β-CG + NAC group were orally administrated with 50 mg (kg BW)^−1^ NAC for 3 days, while pigs in the other two groups were orally administrated with the same volume of sterile saline. NAC numerically reduced diarrhea incidence (− 46.2%) and the concentrations of hydrogen peroxide and malondialdehyde, but increased claudin-1 and intestinal fatty-acid binding protein (iFABP) protein abundances and activities of catalase and glutathione peroxidase in the jejunum of β-CG-challenged piglets. Although β-CG challenge decreased the villus height, villus height/crypt depth ratio, and mRNA levels of *claudin-1* and *occludin*, no significant differences were observed in these indices between the control and β-CG + NAC groups, suggesting the positive effects of NAC supplementation on intestinal mucosal barrier function. Moreover, NAC increased the concentrations of citrulline and D-xylose in the plasma, as well as the expression of genes for aquaporin (*AQP*) 3, *AQP4*, peptide transporter 1 (*PepT1*), sodium/glucose co-transporter-1 (SGLT-1), potassium inwardly-rectifying channel, subfamily J, member 13 (*KCNJ13*), and solute carrier family 1 member 1 (*SLC1A1*) in the jejunum, demonstrating that NAC augmented intestinal metabolic activity and absorptive function. Remarkably, NAC decreased Atg5 protein abundance and the LC3II/LC3I ratio (an indicator of autophagy) in the jejunum of β-CG-challenged piglets. Taken together, NAC supplementation improved intestinal function and attenuated intestinal autophagy in β-CG-challenged piglets.

## Introduction

Soybean is a high-quality protein source, and possesses good nutritional, processing, and functional properties^1^. Therefore, soybeans are major ingredients of some infant formulas. However, soybeans contain allergenic substances, which can induce diarrhea and intestinal inflammation, therefore greatly restricting the use of soybean products in the diets of young mammals, particularly human infants, calves, and weanling pigs^2,3^. Among anti-nutritional factors (ANFs) in soybeans, including β-conglycinin (β-CG), glycinin, agglutinin, and trypsin inhibitor, β-CG has been identified as one of the major allergens^4,5^. Emerging evidence shows that soybean β-CG causes intestinal abnormalities, including inflammation^6^, oxidative stress^7^, mucosal barrier dysfunction^8^, defects of nutrient absorption^9^, and enterocyte damage^10^. Due to these adverse effects, strategies are being employed to reduce or inactivate the soybean β-CG, such as heating, pressurizing, fermentation, enzymatic hydrolysis, and genetic modifications. However, these strategies always elevate the price of soy products and yet could not completely eliminate β-CG. Therefore, any nutritional measures, which can alleviate the intestinal damage induced by soybean β-CG, would be effective strategies to increase the use of soy products in the diets of young animals.

Recently, we have reported that β-CG enhances autophagy activity in vitro by observing the enhanced eGFP-LC3 puncta and LC3-II/LC3-I in enterocytes^11^. Autophagy is extensively involved in various cellular processes associated with physiological and pathological conditions^12^. It is of great interest to know whether autophagy could involve in the pathological process triggered by soybean allergenic proteins. Unfortunately, the literature regarding autophagy induced by soybean allergic proteins is scare. Under stress conditions, excessive reactive oxygen species (ROS) will be generated, thereby promoting the activation of autophagy, apoptosis and necrosis^13^. Given the oxidative stress caused by β-CG administration^7^, we proposed that ROS play a critical role in elevating autophagy activity in enterocytes. Therefore, ROS scavengers, such as antioxidants, may be conducive to attenuate the adverse effects of β-CG on intestinal function in animals.

*N*-acetylcysteine (NAC), a potent antioxidant, is a precursor of L-cysteine, which is utilized for the synthesis of reduced glutathione. Clinical studies have shown that the efficacy of NAC in the treatment of clinical disease results mainly from its antioxidant or radical scavenger properties^14^. Moreover, NAC is a promising bioactive substance for various diseases, such as gastrointestinal inflammation, cardiac injury, acute respiratory distress syndrome, bronchitis, AIDS, nephropathy, and psychiatric disorders^15^. These actions of NAC are associated with detoxifying the reactive metabolites of acetaminophen, altering dopamine release, restoring mitochondrial function, reducing ROS generation, and exerting an anti-inflammatory effect. Using a porcine or chicken model of intestinal dysfunction induced by lipopolysaccharide or heat stress, we also found that dietary NAC supplementation could improve intestinal function and animal health^16,17^. Intriguingly, NAC has also been reported to enhance enterocyte growth and protein synthesis independent of glutathione production^18^. Therefore, NAC may act through multiple mechanisms to protect the gut from inflammatory and oxidative injuries. In light of the foregoing, this study was conducted to test the hypothesis that NAC may improve intestinal function and attenuate autophagy in piglets challenged with β-CG.

## Results

### Body weight and diarrhea incidence

**Figure 1:**
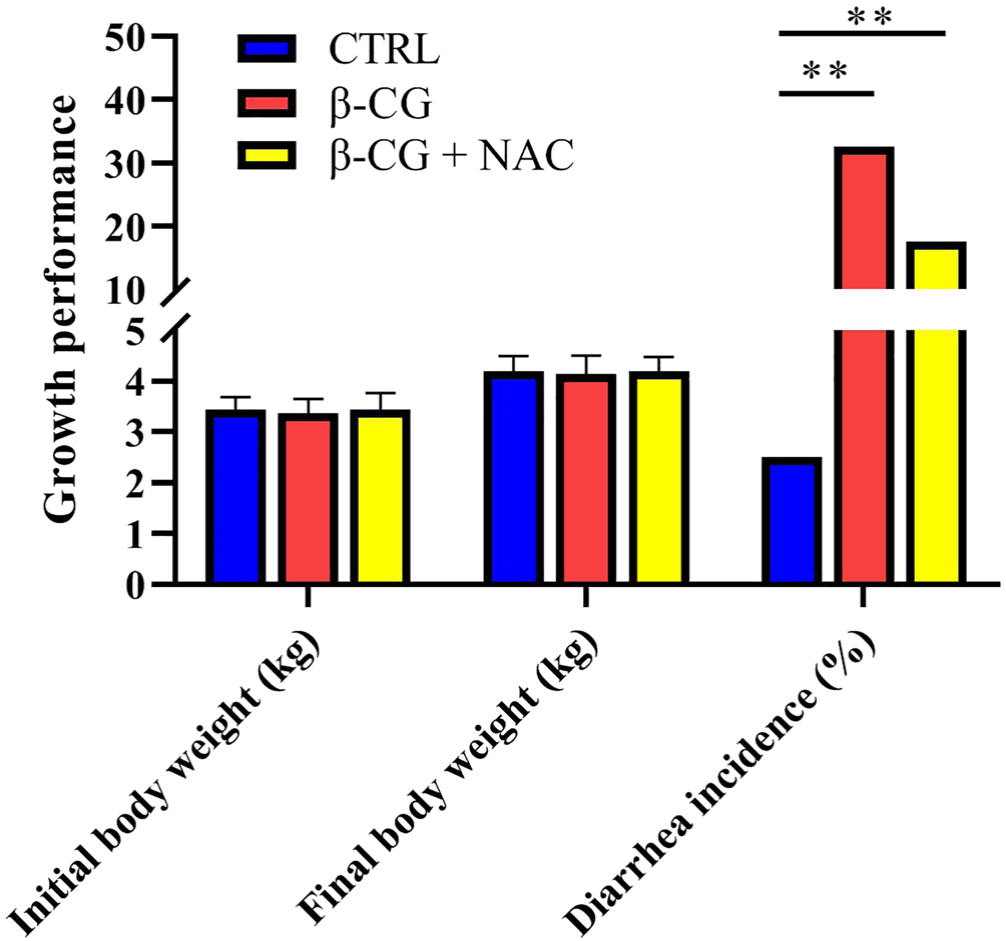
Growth performance and diarrhea incidence of piglets. Piglets in the control group were fed a liquid diet containing 10% casein, whereas those in the β-CG and β-CG + NAC groups were fed liquid diets containing 9.5% casein and 0.5% β-CG for 2 days. Thereafter, pigs in the β-CG + NAC group were orally administrated with 50 mg (kg BW)^−1^ NAC for 3 days, while those in the other two groups were orally administrated with the same volume of sterile saline. Body weights of piglets were recorded and the incidence of diarrhea was observed 3 times per day. Data are means ± SEM, n = 8. ^**^P < 0.01.

Data on the body weight and diarrhea incidence of piglets are presented in Fig. 1. There was no significant difference in final body weight among the three groups of piglets. However, β-CG challenge increased (*P* < 0.05) the incidence of diarrhea in piglets, compared with the control group. Administration of 50 mg (kg BW)^−1^ NAC numerically decreased the incidence of diarrhea by 46.2% (*P* = 0.121) after β-CG challenge.

### Plasma indices

**Figure 2:**
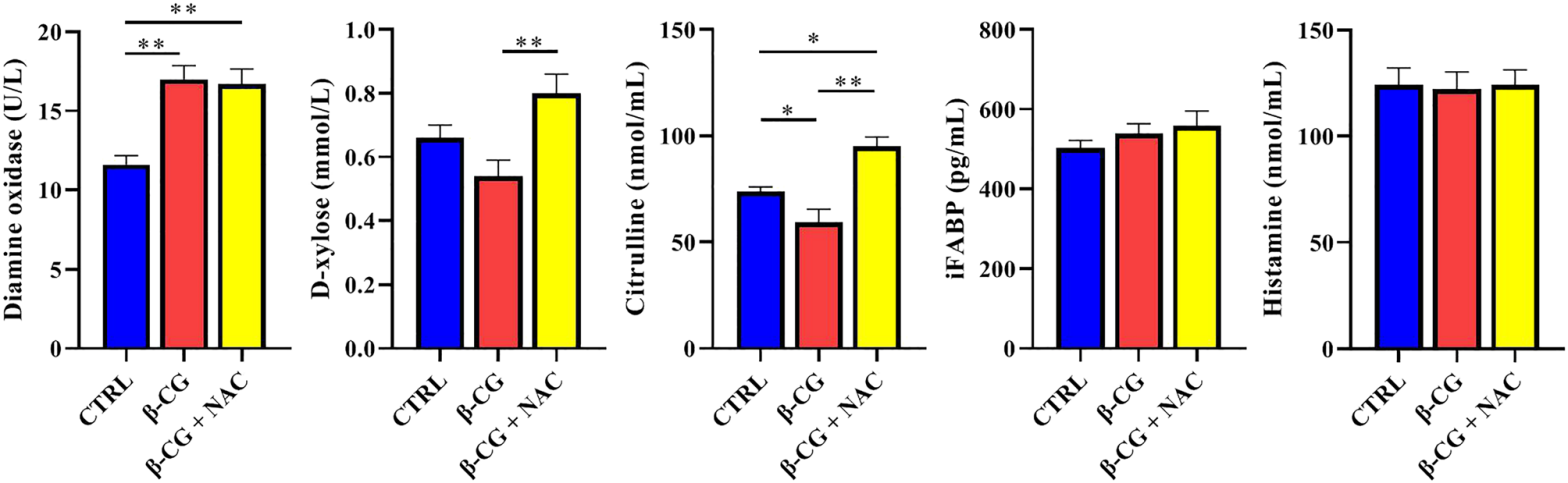
Measurement of plasma indices in piglets. D-xylose and diamine oxidase in plasma were determined by using commercially available kits. Concentrations of histamine and citrulline in plasma were determined by high-performance liquid chromatography. Level of iFABP in the plasma was measured by using an iFABP ELISA kit. Data are means ± SEM, n = 8. ^*^P < 0.05, ^**^P < 0.01.

As shown in Fig. 2, compared with the control group, β-CG challenge reduced (*P* < 0.05) citrulline concentration, but increased (*P* < 0.05) DAO activity, in the plasma of piglets. However, NAC elevated (*P* < 0.01) the plasma levels of citrulline and D-xylose of β-CG-challenged piglets. Additionally, no differences in plasma concentrations of iFABP or histamine were observed among the three groups of pigs.

### Antioxidative enzymes and related products

**Figure 3:**
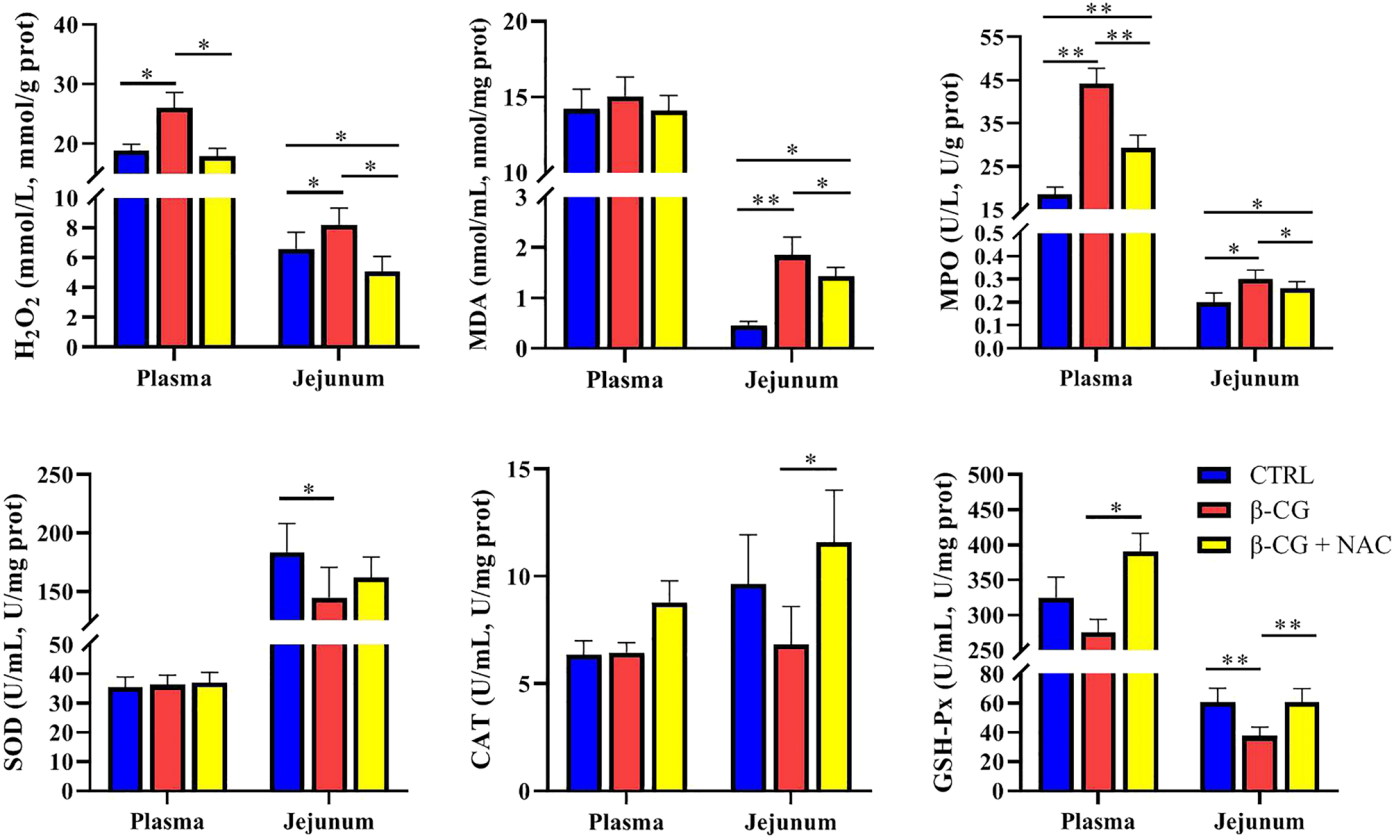
Oxidants and antioxidant proteins in the plasma and jejunal mucosa of piglets. Activities of superoxide dismutase (SOD), catalase (CAT), and glutathione peroxidase (GSH-Px), as well as the concentrations of H_2_O_2_, MDA, and MPO in the plasma and jejunal mucosa were determined by using commercially available kits. Data are means ± SEM, n = 8. ^*^P < 0.05, ^**^P < 0.01.

In order to determine the effects of NAC on redox status of β-CG piglets, the antioxidative enzymes and related products were measured in the plasma and jejunum (Fig. 3). Compared with the control group, β-CG challenge elevated the concentrations of H_2_O_2_ (plasma and jejunum), MDA (jejunum) and MPO (plasma and jejunum), but reduced the activities of jejunal SOD and GSH-Px in piglets. However, dietary NAC administration decreased the levels of H_2_O_2_ (plasma and jejunum), MDA (jejunum) and MPO (plasma and jejunum), but increased the activities of CAT (jejunum) and GSH-Px (plasma and jejunum) in piglets, compared with the β-CG group.

### Intestinal morphology

**Figure 4:**
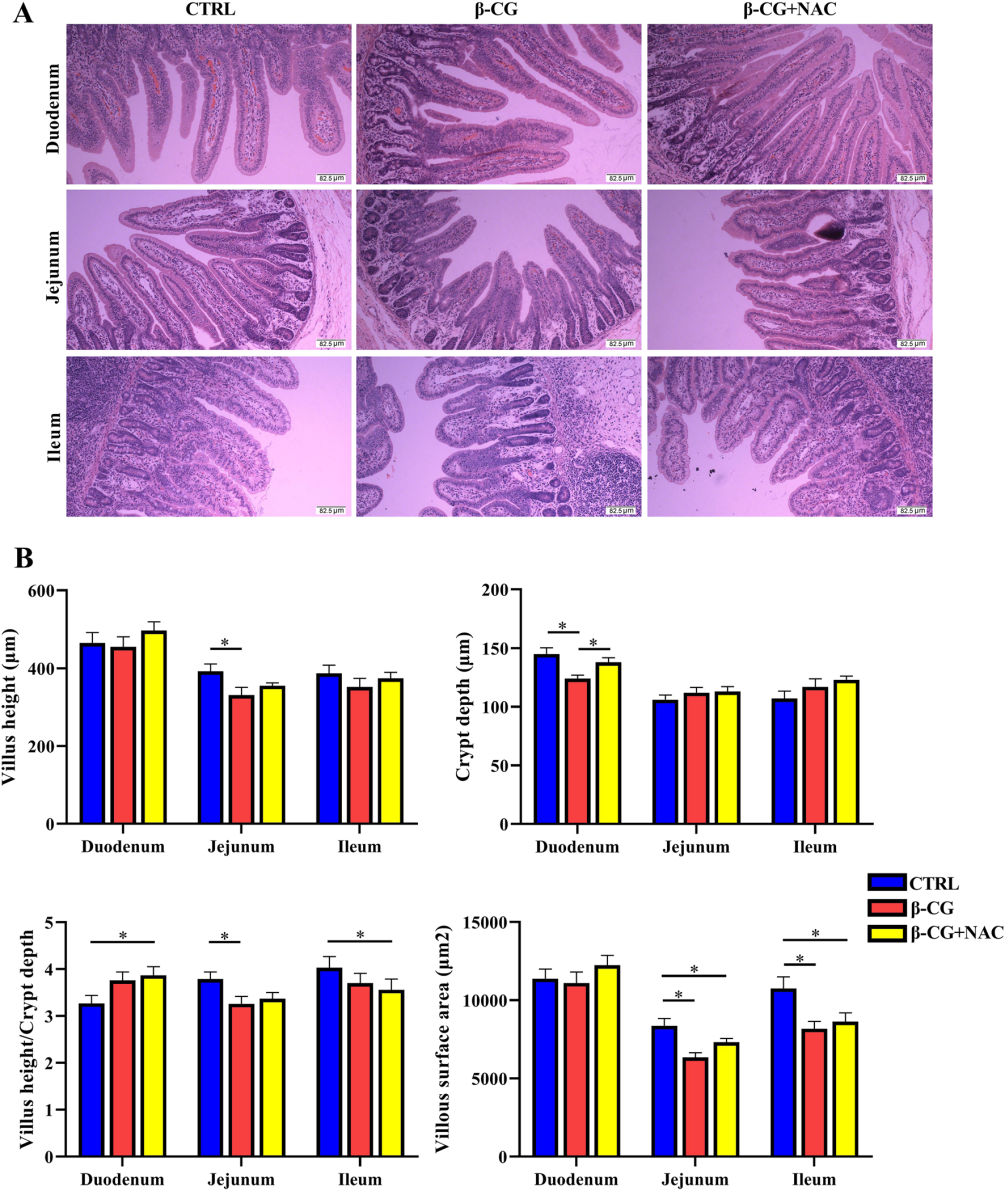
Intestinal morphology in piglets. (**A**) Sections of intestinal segments were stained with haematoxylin and eosin (× 100). (**B**) Villus height is defined as the distance from the villus tip to crypt mouth, and crypt depth is the depth of the distance from the crypt mouth to the base, and villus width is the width at half of villus height. Villous surface area (VSA) was calculated according to the equation: VSA = 2πrh (r = villus width/2, h = villus height). Data are means ± SEM, n = 8. ^*^P < 0.05.

Data on the small-intestinal morphology are summarized in Fig. 4. Compared to the control group, piglets in the β-CG group exhibited reductions (*P* < 0.05) in crypt depth (CD) in the duodenum, villus height (VH) and VH/CD ratio in the jejunum, and villous surface area in the jejunum and ileum. However, no significant differences were observed in the jejunal VH or the VH/CD ratio between the control and β-CG + NAC groups, indicating that NAC treatment attenuated the decreases in the jejunal VH and VH/CD ratio.

### Apoptotic cells in the jejunum

**Figure 5:**
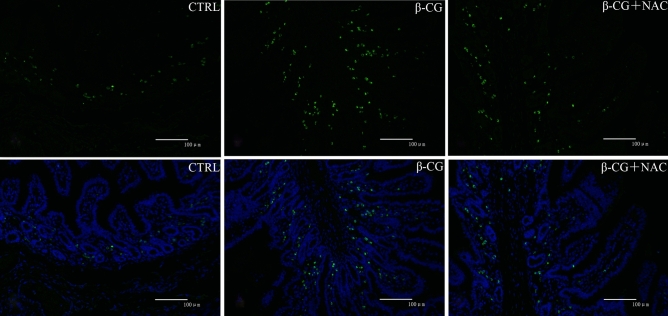
Apoptotic changes in the jejunal mucosa as assessed by the TUNEL method. Paraffin sections of the jejunum were double stained for TUNEL (green) and DAPI (blue) assays. β-CG induced an increase in enterocyte apoptosis in the jejunum of piglets, and this effect of β-CG was alleviated by NAC treatment (× 200).

Apoptotic cells in the jejunum of piglets were determined by the TUNEL method. The green dots in the pictures represent the apoptotic cells (Fig. 5). Of note, β-CG challenge enhanced enterocyte apoptosis, which was attenuated by NAC administration.

### Gene expression in the jejunum

**Figure 6:**
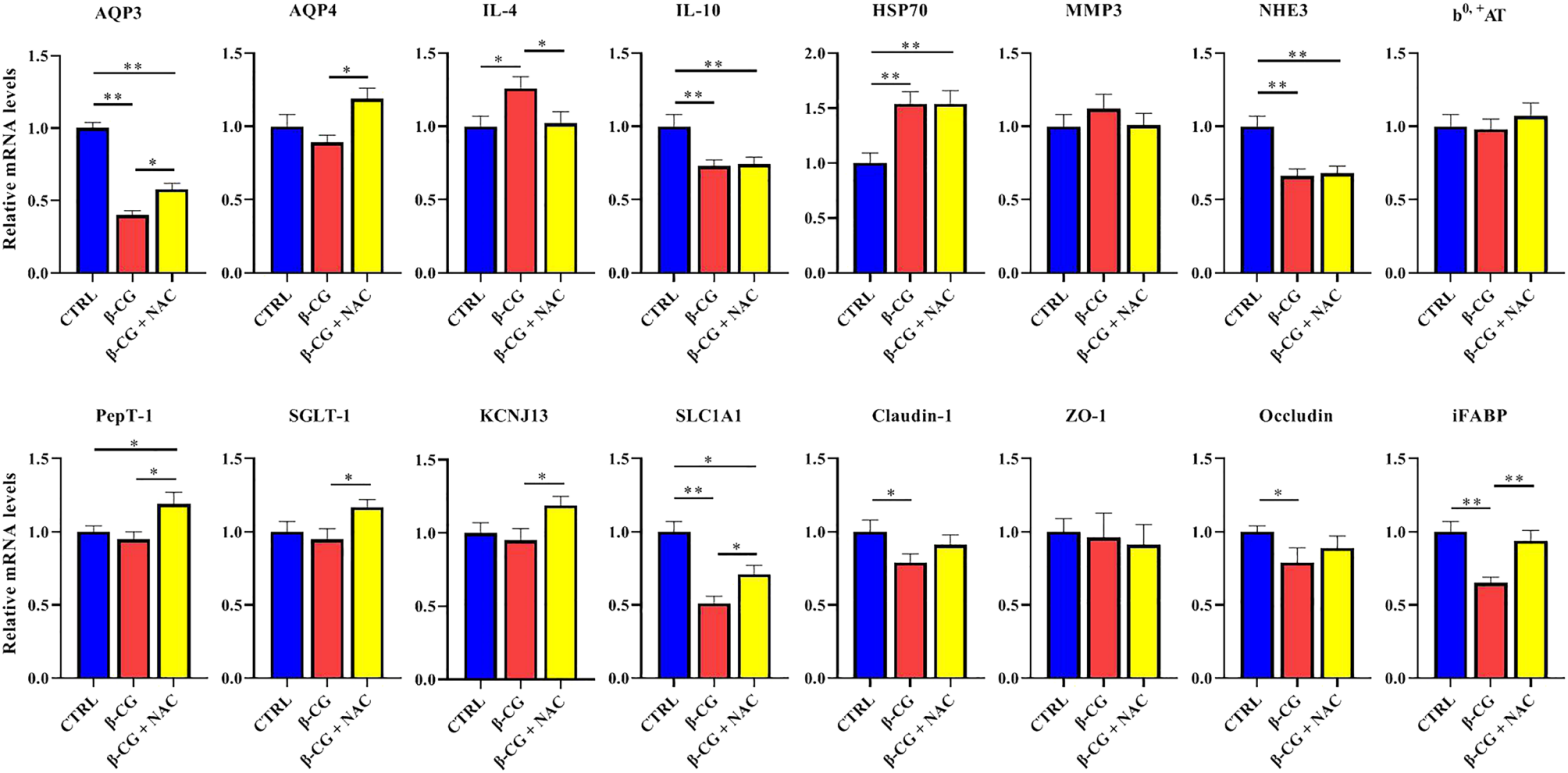
mRNA levels of genes in the jejunum of piglets. Total RNA was extracted from the jejunal mucosa and was analyzed for mRNA levels of genes by real-time RT-PCR with the use of specific primers. RPL4 was used as an internal control. All mRNA levels in the control group were regarded as 1. Data are means ± SEM, n = 8. ^*^P < 0.05, ^**^P < 0.01.

Both NAC and β-conglycinin affected the expression of intestinal genes in piglets (Fig. 6). In the jejunum, β-CG dramatically down-regulated (*P* < 0.01) the expression of *AQP3*, *IL-10, NHE3*, *SLC1A1*, *Claudin-1*, *Occludin*, and *iFABP*, but up-regulated (*P* < 0.05) the expression of *IL-4* and *HSP70* in piglets. Administration of NAC decreased the *IL-4* mRNA level, but increased (*P* < 0.05) the jejunal mRNA levels for *AQP3*, *AQP4*, *PepT-1*, *SGLT-1*, *KCNJ13*, *SLC1A1* and *iFABP* in comparison with the β-CG group. Additionally, NAC supplementation attenuated the reductions in the mRNA levels for jejunal *claudin-1* and *occludin* of β-CG-challenged piglets based on the finding that there were no significant differences in the expression of these two genes between the control and β-CG + NAC groups.

### Protein expression in the jejunal mucosa

**Figure 7:**
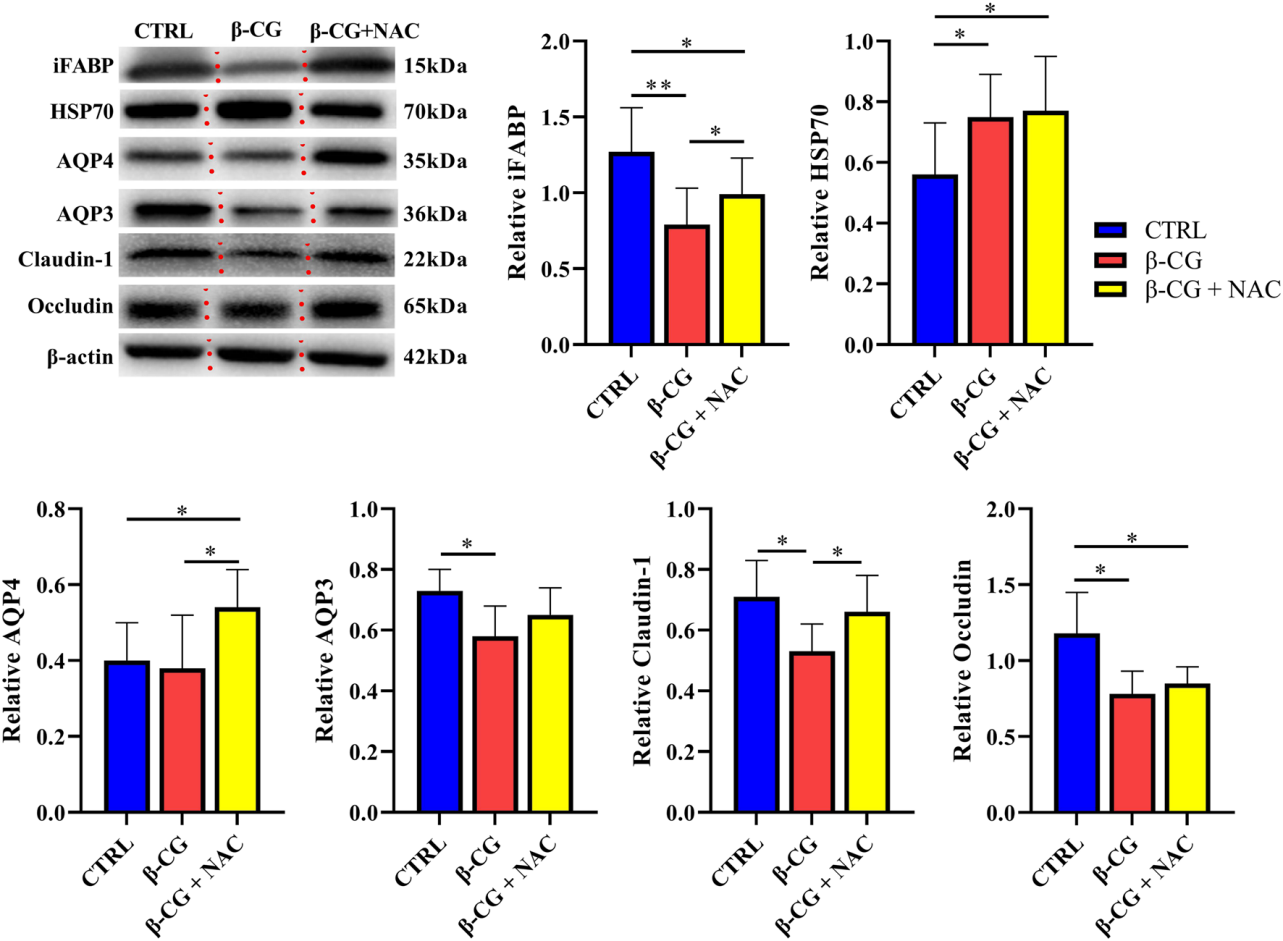
Protein abundances of AQP3, AQP4, iFABP, claudin-1, occludin, and HSP70 in the jejunal mucosa of piglets (full length blots are shown in Supplementary Figure S1–S6). Approximately 100 mg of jejunal mucosal samples were powdered and homogenized in lysis buffer. After centrifugation, the mucosal samples were separated by 7.5% (for occludin and HSP70), 10% (for β-actin and claudin-1), or 12% (for iFABP, AQP3 and AQP4) SDS-PAGE and electroblotted onto the polyvinylidene difluoride membrane. Thereafter, membranes were incubated with a primary antibody and a secondary antibody as described in MATERIALS AND METHODS. Data are mean ± SEM, n = 8. ^*^P < 0.05, ^**^P < 0.01.

**Figure 8:**
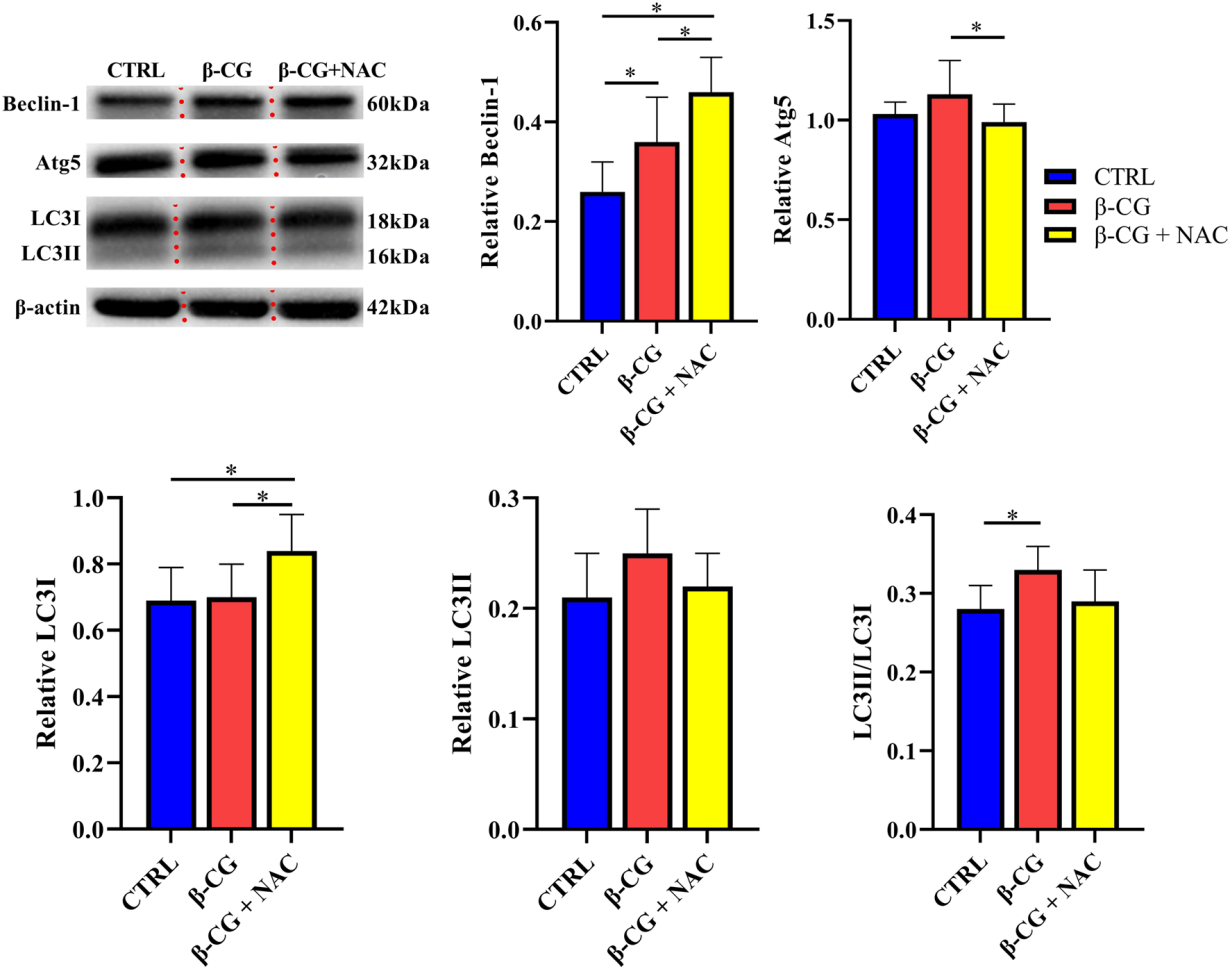
Protein abundances of beclin-1, Atg5 and LC3 (LC3I and LC3II) in the jejunal mucosa of piglets (full length blots are shown in Supplementary Figure S7-S10). Approximately 100 mg of jejunal mucosal samples were powdered and homogenized in lysis buffer. After centrifugation, the mucosal samples were separated by 10% (for β-actin) or 12% (for Atg 5, beclin-1 and LC3) SDS-PAGE and electroblotted onto polyvinylidene difluoride membrane. Thereafter, membranes were incubated with a primary antibody and a secondary antibody as described in MATERIALS AND METHODS. Data are mean ± SEM, n = 8. ^*^P < 0.05.

As shown in Figs. 7 and 8, β-CG challenge increased (*P* < 0.05) the abundance of beclin-1 and HSP70 proteins, and the LC3II/LC3I ratio, while decreasing (*P* < 0.05) the abundance of AQP3, iFABP, claudin-1, and occludin proteins in the jejunum of piglets. However, NAC treatment significantly increased (*P* < 0.05) the abundance of AQP4, iFABP, claudin-1, Beclin-1, and LC3I proteins, but decreased (*P* < 0.05) the abundance of the autophagy-related protein 5 (Atg5) protein in the jejunum of piglets challenged with β-CG. Notably, no significant differences were observed in the protein abundances of AQP3 or LC3II/LC3I ratio in the jejunum between the control and β-CG + NAC groups of piglets (Figs. 7 and 8), indicating that NAC attenuated the decreases in these two variables in β-CG-challenged piglets.

## Discussion

Allergenic proteins and anti-nutritional factors in soybeans are well known to depress growth, impair the integrity of the intestine, and induce allergies in young animals^19–21^. However, the mechanisms underlying the adverse effects of the allergens remain largely unknown. In the present study, we determined the effects of β-CG, one of the major allergenic proteins in soybeans, on growth performance, intestinal function and intestinal autophagy activity in neonatal piglets. In addition, we assessed whether NAC treatment could attenuate the β-CG-induced adverse effects in pigs. Our results showed that β-CG challenge significantly increased the diarrhea incidence, and NAC supplementation numerically reduced diarrhea incidence. This indicates that NAC could exert positive effects on intestinal health in piglets after β-CG challenge. Consistently, we have reported the beneficial effects of NAC on intestinal function in piglets challenged with lipopolysaccharide (LPS) and in broilers exposed to heat stress^16,17^. Unexpectedly, neither β-CG nor NAC affected the growth performance of piglets during the short experimental period. Previous studies have demonstrated that dietary supplementation with glycinin (> 4%, 10 days) or β-CG (4%-8%, 3 days) can reduce average daily gain in piglets^19,21^. Therefore, the discrepancy between our and the previous studies may be attributed to the different dosage and the different duration of β-CG challenge. On the other hand, short-term NAC supplementation may not affect the growth performance of piglets irrespective of stress^17,22–24^, and the effect of NAC on piglets may differ from that on birds^16,25^.

The function of the small intestine includes not only the digestion and absorption of nutrients, but also the establishment of a physical and immunological barrier against foreign antigens. In pigs, the jejunum is the main site of nutrient digestion and absorption, whereas the jejunum and the ileum are more sensitive than other segments of the gut to antigens^26^. Moreover, we found that the villus height, villus heght/crypt depth, and villous surface area of the jejunum were significantly affected by β-CG challenge (Fig. 4). Therefore, the jejunum was used for the analysis of gene and protein abundances in the present study. β-CG is known to induce intestinal hypersensitivity^6^. In the present study, jejunal *IL-4* and HSP70 were up-regulated, whereas *IL-10* was down-regulated in response to β-CG challenge. However, NAC supplementation restored the IL-4 expression in the jejunum of β-CG-challenged piglets to the normal level, but did not affect the jejunal *IL-10* and HSP70 expression or plasma histamine concentration in β-CG + NAC piglets. IL-4 and IL-10 are produced by Th2 cells^27^ and are involved in the production of antibody IgE, which can further induce mast cells to release active mediators, such as histamine and cytokines, resulting in hypersensitivity^28^. The expression of *IL-4* and *IL-10* could be differentially regulated by β-CG (Fig. 6). Therefore, because no significant difference was observed in plasma histamine concentrations among the three groups of piglets, it is uncertain whether β-CG challenge induced a hypersensitivity of piglets. In the contrary, β-CG challenge consistently induced oxidative stress in the jejunum based on the reductions in the activities of antioxidative enzymes and the elevations in HSP70 expression and oxidants, such as H_2_O_2_ and MDA. Since NAC can protect cells from oxidative damage through direct (by reacting with oxidants) and indirect (by increasing GSH-Px) ways^16^, we proposed that NAC could attenuate the intestinal oxidative stress induced by β-CG. Additionally, as shown in Fig. 3, although NAC treatment significantly decreased MPO and MDA concentrations compared with the β-CG group, NAC did not restore the values in the plasma and jejunum of β-CG piglets to normal levels. We have previously demonstrated that dietary NAC (about 50 mg/kg BW, 21 days) could restore jejunal MDA in lipopolysaccharide-challenged piglets to normal levels^29^. Similarly, Zhu et al. have reported that NAC (about 70 mg/kg BW, 12 days) restored serum MDA in weaned piglets to normal levels^30^. In the present study, the lack of an effect of NAC on normalizing MDA and MPO concentrations in the jejunum and plasma of β-CG piglets is possibly due to the lower dosage (50 mg/kg BW) and a short period of NAC treatment (3 days).

Intestinal morphology is closely associated with the intestinal integrity and mucosal barrier function. Allergenic proteins in soybeans commonly induce inflammatory disorders in the small intestine, involving villous atrophy, as well as enterocyte proliferation, apoptosis, and migration^8,20,31^. Consistent with the results of these studies, β-CG challenge enhanced enterocyte apoptosis and decreased the jejunal VH and VH/CD ratio. However, no significant differences were observed in the jejunal VH or VH/CD ratio between the control and β-CG + NAC groups, indicating that these adverse effects could be mitigated by NAC supplementation. The reason why NAC inhibits β-CG-induced the apoptosis of intestinal epithelial cells (Fig. 5) is unclear. An in vitro study revealed that addition of NAC to cell culture medium could enhance enterocyte growth and protein synthesis^18^. Moreover, although NAC administration did not alter the levels of plasma DAO and iFABP, both of which may serve as biomarkers of gut mucosal injury^32,33^. Additionally, the abundance of iFABP in the small intestine was significantly increased by NAC supplementation in β-CG-challenged pigs, further substantiating the notion that NAC can stimulate global and specific syntheses of proteins by enterocytes. Of note, NAC may regulate iFABP expression in enterocytes at both transcriptional and translational levels since the abundances of jejunal iFABP mRNA and protein were augmented in β-CG-challenged piglets that received NAC administration. Furthermore, NAC may also protect the intestinal barrier function in piglets because its supplementation attenuated the decreases in jejunal claudin-1 protein abundance and jejunal mRNA levels for *claudin-1* and *occludin* in β-CG-challenged piglets. By using the intestinal injury model of LPS-challenged piglets, we have reported the elevations in protein abundances of jejunal occludin and claudin-1 by NAC administration^17^. Collectively, NAC treatment could improve intestinal integrity and function in piglets challenged by β-CG.

In healthy pigs, D-xylose is readily absorbed by the small intestines. However, under conditions of malabsorption, the entry of D-xylose from the intestinal lumen to the portal vein is impaired, and therefore the concentrations of D-xylose in both blood and urine are reduced^34^. Moreover, Papadia et al*.*^35^ reported that the plasma concentration of citrulline (which is exclusively synthesized from glutamine and proline by enterocytes in most mammals including humans and pigs) was a reliable marker for the mass and metabolic activity of the small bowel in short bowel patients. Therefore, plasma D-xylose and citrulline levels can serve as indicators of the ability of the small intestine to absorb nutrients^34–36^. Our results that NAC increased plasma citrulline concentrations and attenuated the decrease in D-xylose concentrations in the plasma of β-CG-challenged piglets indicated improvements in intestinal mass/metabolic activity and absorptive function in response to oral administration of NAC. Certainly, the precise changes in absorptive function needs to be measured in further applied studies. By using porcine models of intestinal injury induced by LPS challenge and porcine epidemic diarrhea virus (PEDV) infection, we also found that dietary NAC supplementation increased blood D-xylose concentration and the intestinal absorptive function^17,22^.

Another important finding of the present study is that supplementation with NAC may enhance intestinal water absorption by stimulating the expression of water transporters (such as AQP3, AQP4, and NHE3) in β-CG-challenged piglets. This, in turn, reduces the incidence of diarrhea in piglets. Aquaporins (AQPs) are small integral membrane proteins that provide channels for the transport of water across the cell membrane^37^. AQP3 is expressed abundantly in the luminal side of the intestinal mucosa^38^, and is expressed more abundantly than AQP4^39^. Emerging evidence shows that intestinal AQP3 and AQP4 are down-regulated in animals with diarrhea^40–42^. Similar results were observed in the present study, although the underlying mechanisms remain to be identified. Sodium hydrogen exchanger 3 (NHE3) is one of the five plasma membrane Na^+^/H^+^ exchangers and works in conjunction with SLC26A3, a key intestinal epithelial Cl^−^/HCO3^−^ exchanger. Both NHE3 and SLC26A3 were reported to play a crucial role in mediating intestinal fluid and sodium absorption, and were down-regulated in infectious diarrhea^43,44^. In agreement with those studies, a decrease in intestinal *NHE3* mRNA levels was observed in β-CG piglets with higher diarrhea incidence. Because jejunal *NHE3* mRNA levels did not differ between the β-CG and β-CG + NAC groups, the up-regulated expression of intestinal AQP3 and AQP4 by NAC may preferentially facilitate intestinal water absorption. In this regard, it is noteworthy that NAC increased the expression of genes encoding for amino acid and peptide transporters (SLC1A1 and PepT1), a sodium-glucose co-transporter (SGLT-1), and a potassium channel named potassium inwardly-rectifying channel, subfamily J, member 13 (KCNJ13) (Fig. 6). This further supports our notion that NAC improved the intestinal absorptive function in β-CG challenged piglets.

The last but salient finding of this study is that NAC may regulate intestinal autophagy as indicated by decreases in both the abundance of the Atg5 protein and the LC3II/LC3I ratio in the jejunum of β-CG-challenged piglets. Autophagy is a catabolic process involving the degradation of the cell’s own constitutive, injured, and aged proteins and organelles for adaptation and survival^45^. However, excessive autophagy leads to cell death^46^. There are several biomarkers of autophagy process. LC3 (microtubule-associated protein light chain 3B), a marker of autophagosomes, is useful in biochemical assays to assess autophagosome numbers^12^. LC3II/LC3I is a quantitative marker for monitoring autophagy^47^. Atg5 is associated with the ATG12-ATG16 complex that is necessary for the conjugation of LC3-I to phosphatidylethanolamine to form LC3-II (LC3-phosphatidylethanolamine conjugate). Beclin-1 is a mammalian ortholog of the yeast autophagy-related gene 6 (Atg6) and interacts with the class III PI3-kinase signalling complex to positively control the formation of autophagic vacuoles^48,49^. It is interesting that β-CG challenge increased the Beclin-1 expression and the LC3II/LC3I ratio, indicating the enhancement of intestinal autophagy activity by β-CG challenge. This finding provides an important clue into the underlying mechanism whereby β-CG exerts adverse effects, although alteration in autophagic activity needs to be directly measured in future studies. Yi et al*.*^11^ reported that β-CG enhanced enterocyte autophagy in vitro. To date, there is little information on the relationship between β-CG and intestinal autophagy. Because ROS are important factors to activate autophagy^13^, an increase in its activity may be partially attributed to excessive ROS in the β-CG piglets as indicated by the increased levels of H_2_O_2_ and MDA in the jejunum. As a potent antioxidant, NAC inhibits excessive autophagy possibly through scavenging ROS and attenuating the oxidative stress in the small intestine (Fig. 3). Other actions of NAC on autophagy may be mediated through enhancing the expression of anti-apoptotic protein (Bcl-2)^50^, regulating inflammation^51^, and activating the mammalian target of rapamycin (mTOR) signalling pathway^52^. Therefore, further studies are warranted to elucidate the mechanisms responsible for the β-CG-induced intestinal autophagy and its intervention by NAC.

## Conclusions

Results of this study showed that NAC improved intestinal morphology, antioxidative capacity, absorptive ability, and mucosal integrity in β-CG-challenged piglets. Importantly, our findings indicated a role for autophagy in the regulation of intestinal function by NAC. Collectively, the current work provides a new perspective for understanding nutritional interventions of intestinal diseases in pigs, and also has important implications for improving formulas for human infants, piglets and other mammals.

## Materials and methods

### Preparation of β-CG

β-CG was isolated from low-temperature defatted soy flour by the method of Nagano et al*.*^53^. The purity of β-CG was determined by SDS-PAGE analysis and β-CG content in the globulins was greater than 80%.

### Experimental animals and design

The experimental procedures involving animals for the current study were carried out in accordance with the Chinese Guidelines for Animal Welfare and Experimental Protocol, and were approved by the Animal Care and Use Committee of Wuhan Polytechnic University. Twenty-four crossbred healthy female piglets (Duroc × Landrace × Yorkshire) reared by sows were weaned at 7 ± 2 days of age and were housed in a temperature-controlled nursery barn (28–30 °C). After a 5-day period of adaptation, piglets (12 ± 2 days of age, average body weight of 3.44 ± 0.28 kg; d 0 of the trial) were randomly allotted to one of three treatment groups: control, β-CG, and β-CG + NAC groups. Each treatment had eight piglets. Piglets were fed a liquid diet that contained milk replacer (containing hydrolysed wheat protein rather than soybean protein) and casein as protein sources (Table 1). The liquid diet was formulated according to the ratio of diet/water (1:4)^22^. During the 5-day period of adaptation, all piglets were fed a liquid diet containing 10% casein. At day 0 of the trial, piglets in the control group were continuously fed the same diet, whereas those in the β-CG and β-CG + NAC groups were fed a liquid diet containing 9.5% casein and 0.5% β-CG for 2 days. At day 2 of the trial, pigs in the β-CG + NAC group were orally administrated with 50 mg (kg BW)^−1^ NAC, while pigs in the other two groups were orally administrated with the same volume of sterile saline for 3 days. In order to exclude the possible effects of β-CG-induced food intake reduction on the intestinal indices of piglets, the control and β-CG + NAC piglets were fed the same amounts of liquid diet as the β-CG piglets. The dosage of β-CG was chosen according to the result of our preliminary study in which the piglets fed a liquid diet containing 1% or 2% β-CG exhibited severe diarrhea and a depression of feed intake, while those fed a liquid diet containing 0.5% β-CG showed a slight reduction in feed intake and a moderate increase in diarrhea incidence. The dosage of NAC was chosen in the present study in accordance with our previous work^22^. The current experiment lasted for 5 days.

**Table 1:** Ingredient composition and nutrient levels of milk replacer powder diets (as-fed basis).

	CTRL	β-CG	β-CG + NAC
**Ingredients (%)**			
Maize	12.00	12.00	12.00
Milk replacer^a^	39.43	39.40	39.40
Whey powder	20.00	20.00	20.00
Casein	10.00	9.5	9.5
β-Conglycinin	0	0.5	0.5
Flour (wheat)	8.00	8.00	8.00
Spray-dried plasma protein	3.00	3.00	3.00
Lactose	3.00	3.00	3.00
Salt	1.80	1.80	1.80
Calcium phosphate	1.45	1.45	1.45
Premix^b^	1.00	1.00	1.00
L-Lys	0	0.03	0.03
DL-Met	0.22	0.22	0.22
**Nutrient composition**^**c**^			
Digestible energy (MJ/kg)	14.72	14.70	14.70
Crude protein (%)	24.10	24.05	24.05
Lys (%)	1.81	1.83	1.83
Met + Cys (%)	0.97	0.97	0.97
Ca (%)	0.83	0.82	0.82
P (%)	0.80	0.80	0.80

^a^Milk replacer contained hydrolyzed wheat protein rather than soybean protein.

^b^Premix provided the following vitamins and trace minerals per kilogram of milk replacer powder diet: Fe, 100 mg; Cu, 20 mg; Mn, 40 mg; Zn, 100 mg; I, 0.4 mg; Se, 0.3 mg; retinol acetate, 3.6 mg; cholecalciferol_,_ 0.05 mg; DL-α-tocopheryl acetate, 30 mg; Menadione_,_ 3 mg; thiamin_,_ 2 mg; riboflavin_,_ 6 mg; pyridoxine_,_ 7 mg; Cyanocobalamin_,_ 0.04 mg; biotin, 0.1 mg; Folic acid, 2 mg; niacin, 40 mg; D-calcium pantothenate, 20 mg.

^c^Analyzed value.

Body weights of piglets were recorded every day and the incidence of diarrhea was observed 3 times per day. Fecal scores were determined according to stool conditions: 0, firm and shaped; 1, pasty; 2, semiliquid; and 3, liquid^54^. The occurrence of diarrhea was defined as the maintenance of a fecal score at 2 or 3 for 2 consecutive days. Then, the incidence of diarrhea was calculated according to the formula as reported by Liu et al. (2008): incidence of diarrhea = total number of pigs with diarrhea/(total number of pigs × experimental days) × 100%, where “total number of pigs with diarrhea” was defined as the sum of the number of pigs with diarrhoea observed each day^55^.

### Sample collection

On day 5 of the trial, d-xylose was orally administrated to all piglets at the dose of 0.1 g (kg BW)^−1^ to test the absorptive capacity of the small intestine^56^. At 1 h after d-xylose administration, blood samples were collected from the anterior vena cava into heparinized vacuum tubes and centrifuged (2000*g* for 15 min at 4 °C) to obtain plasma^22^. Plasma samples were stored at − 80 °C until analysis.

Piglets were killed under anaesthesia with an intravenous injection of pentobarbital sodium [80 mg (kg BW)^−1^]. The pig abdomen was then opened immediately from the sternum to the pubis, and the whole gastrointestinal tract was immediately exposed. Intestinal segments (2 cm and 10 cm in length) were obtained from the distal duodenum, mid-jejunum, and mid-ileum^57^, and then flushed with saline to remove intestinal contents. One sample of each intestinal tissue, about 2 cm in length, was fixed in 4% paraformaldehyde. Another part of a 10-cm section was opened longitudinally and flushed with pre-cooled PBS, and the intestinal mucosae were then scraped into sterile tubes by using a sterile glass microscope slide at 4 °C^22^. The mucosal samples were rapidly frozen in liquid nitrogen and then stored at − 80 °C until analysis.

### Diamine oxidase (DAO) activity, d-xylose, histamine and citrulline concentrations in the plasma

D-xylose and diamine oxidase (DAO) in plasma were determined by using commercially available kits (Jiancheng Institute of Biological Technology, Nanjing, China). Concentrations of citrulline and histamine in plasma was determined by high-performance liquid chromatography (HPLC) as described by Wu et al*.*^58^ with modifications. Briefly, 50 μL plasma was mixed with 50 μL of 1.5 M HClO_4_ in a tube, followed by addition of 1.125 ml H_2_O and 25 μL of 2 M K_2_CO_3_. The supernatant fluid was collected after centrifugation (10,000*g* for 1 min). In a tube, 100 μL of 1.2% benzoic acid and 1.4 ml H_2_O were mixed with 100 μL of the sample, and the solution was filtered through a 0.22-μm filter cartridge into a 4-ml glass vial. Citrulline was determined by using an HPLC method involving precolumn derivatization with o-phthaldialdehyde (OPA). The latter was prepared by dissolving 50 mg OPA in 1.25 mL methanol, followed by addition of 11.2 mL of 40 mM sodium borate buffer, 50 μL of 2-mercaptoethanol, and 0.4 mL of Brij-35. The HPLC system consisted of a model 2475 multi λ fluorescence detector, a Supelco C18 column (4.6 mm × 15 cm, 3 μm; Sigma-Aldrich, St. Louis, MO, USA) and a Supelco C18 guard column (4.6 mm × 5 cm, 20–40 μm; Sigma Aldrich, St. Louis, MO, USA).

### iFABP in plasma

Intestinal fatty-acid binding protein (iFABP) in the plasma was measured by using an iFABP enzyme-linked immunosorbent assay kit (Hycult Biotech Inc., Frontstraat 2A, UDEN, Netherlands), following the instructions of the manufacturer.

### Antioxidative enzymes and related products in the plasma and jejunal mucosa

Activities of superoxide dismutase (SOD), catalase (CAT), and glutathione peroxidase (GSH-Px), as well as concentrations of hydrogen peroxide (H_2_O_2_), malondialdehyde (MDA), and myeloperoxidase (MPO) in the plasma and jejunal mucosa were determined by using commercially available kits from Nanjing Jiancheng Bioengineering Institute (Nanjing, China). Assays were performed in triplicate.

### Intestinal morphology

After a 24-h period of fixation in 4% paraformaldehyde, the intestinal segments were taken out, and dehydrated using the graded concentrations of ethanol (70% to 100%) and chloroform^56^. After dehydration, the segments were embedded in paraffin, and then placed in a refrigerator to make the paraffin sufficiently hard. Cross-sections of the segments were cut at approximately 5 µm thickness with a microtome and stained with haematoxylin and eosin^56^. In each section, 10 fields were examined using a light microscope with a computer-assisted morphometric system (BioScan Optimetric, BioScan Inc., Edmonds, WA). The villous height (VH), the associated crypt depth (CD), and villous width were measured, and then the ratio of villus height to crypt depth (VH/CD) and villous surface area were calculated. Villus height is defined as the distance from the villus tip to crypt mouth, and crypt depth is defined as the depth of the distance from the crypt mouth to the base, and villus width is defined as the width at half of villus height^59^. All intestinal histological analyses were done by the same person, who was blinded to the treatments.

### Apoptotic changes in the jejunum by the TUNEL method

The TUNEL method was performed to detect the apoptotic changes in the jejunum of piglets as described by Duan et al*.*^60^. Briefly, paraffin sections were deparaffinized in xylene, dehydrated through graded alcohols, and washed in freshly prepared deionized water. Then, the deparaffinized sections were treated with proteinase K for 30 min at 37 ^◦^C and rinsed in PBS. A methanol solution containing 3% hydrogen peroxide was used to block endogenous peroxidase for 5 min. Sections were immersed in a buffer containing terminal deoxynucleotidyl transferase and digoxigenin-labelled nucleotides for 2 h at 37^◦^C. After washing with PBS, sections were incubated with a pre-diluted anti-digoxigenin peroxidase-conjugated antibody for 30 min. Apoptotic cells were detected after the sections were incubated in the 3,3′-diaminobenzidine (DAB) chromogen for approximately 6 min and counterstained with 4′,6-diamidino-2-phenylindole (DAPI). Imaging was performed by using the Olympus IX73 inverted microscope system.

### mRNA analysis by RT-PCR

Frozen intestinal mucosal samples (~ 100 mg) were powdered and homogenized, and total RNA was isolated by using the TRIzol Reagent protocol (Invitrogen Technology, Carlsbad, CA, USA). Total RNA was quantified by using the NanoDrop ND-1000A UV–VIS spectrophotometer (Thermo Scientific, Wilmington, DE, USA) at an OD of 260 nm, and its purity was assessed by determining the OD260/OD280 ratio. Total RNA was reverse-transcribed by using a PrimeScrip RT reagent kit with gDNA Eraser (Takara Bio Inc., Dalian, China) according to the manufacturer’s instruction. cDNA was synthesized and stored at − 20 °C until use.

To amplify cDNA fragments, primer pairs (Table 2) were used for RT-PCR. The RT-PCR was performed by using the SYBR Premix Ex Taq (Takara Bio Inc., Dalian, China) on an Applied Biosystems 7500 Fast Real-Time PCR System (Foster City, CA, USA). The average cycle threshold (Ct) of genes in intestinal samples was determined. Simultaneously, the average Ct of ribosomal protein L4 (RPL4) was determined as the internal reference in each sample to avoid any artifact of variation in the target gene. Results were analyzed by 2^−ΔΔCt^ method^61^. Each biological sample was run in triplicate.

**Table 2:** Sequences of the primers used for quantitative real-time PCR analysis.

Gene	Forward	Reverse
*RPL4*	GAGAAACCGTCGCCGAAT	GCCCACCAGGAGCAAGTT
*AQP3*	AAGCTGTCCCAAGTAAAGCACAA	GCCCTACTTCCTGTTTCACCAC
*AQP4*	TGGTTCTCATCTCCCTTTGCTT	GCGATGCTAATCTTCCTGGTG
*IL-4*	TACCAGCAACTTCGTCCAC	ATCGTCTTTAGCCTTTCCAA
*IL-10*	GGTTGCCAAGCCTTGTCAG	AGGCACTCTTCACCTCCTC
*HSP70*	GACGGAAGCACAGGAAGGA	GAAGACAGGGTGCGTTTGG
*MMP3*	GATGTTGGTTACTTCAGCAC	ATCATTATGTCAGCCTCTCC
*NHE3*	AAGTACGTGAAGGCCAACATCTC	TTCTCCTTGACCTTGTTCTCGTC
*b*^*0,*^^+^*AT*	CGAGTACCCGTACCTGATGGA	TGCGTAGAAGGGCGAAGAA
*PepT-1*	ATTCTCAGGCTCCTTCCAACA	GCAACCCCGCAAACAGA
*SGLT-1*	CCCAAATCAGAGCATTCCATTCA	AAGTATGGTGTGGTGGCCGGTT
*KCNJ13*	ATGGATGTGTCGCTGGTCTTT	CACAACTGCTTGCCTTTACGAG
*SLC1A1*	GGCACCGCACTCTACGAAGCA	GCCCACGGCACTTAGCACGA
*Claudin-1*	GGTGCCCTACTTTGCTGCTC	CCCACACGGTTTTGTCCTTT
*ZO-1*	AGGCGATGTTGTATTGAAGATAAATG	TTTTTGCATCCGTCAATGACA
*Occludin*	TATGAGACAGACTACACAACTGGCGGCGAGTCC	ATCATAGTCTCCAACCATCTTCTTGATGTG
*iFABP*	AGATAGACCGCAATGAGA	TCCTTCTTGTGTAATTATCATCAGT

RPL4: ribosomal protein L4; AQP3: aquaporin 3; AQP4: aquaporin 4; HSP70: heat shock protein 70; IL-4: interleukin 4; IL-10: interleukin 10; MMP3: matrix metallopeptidase 3; NHE3: sodium hydrogen exchanger 3; b^0, +^AT: B(0, +)-type amino acid transporter; PepT-1: peptide transporter 1; SGLT-1: sodium/glucose cotransporter member 1; KCNJ13: potassium voltage-gated channel subfamily J member 13; SLC1A1: excitatory amino-acid transporter 3; ZO-1: tight junction protein 1; iFABP: intestinal fatty-acid binding protein.

### Protein immunoblot analysis

Protein immunoblot analysis was carried out in accordance with the previously described method^62^. Briefly, frozen intestinal mucosal samples (~ 100 mg) were powdered and homogenized in 1 mL of a lysis buffer (BCA Kit from Beyotime Institute of Biotechnology, Jiangsu, China). After centrifugation (12,000*g*, 15 min, 4 °C), the supernatant fluid was aliquoted into micro-centrifuge tubes, to which 2 × SDS sample buffer was added in a 1:1 ratio. The samples were boiled and cooled on ice before use for western blotting. Proteins were separated by electrophoresis on 7.5%, 10% or 12% polyacrylamide gel (depending on the molecular weight of protein), and then electrophoretically transferred to a polyvinylidene difluoride (PVDF) membrane. Skim-milk powder in TBST buffer (1 × Tris-buffered saline including 0.1% Tween 20) was used to block membranes for 2 ~ 2.5 h at 25 °C. Membranes were then incubated with one of the following primary antibodies overnight at 4 °C: AQP3 (1:1000, Santa Cruz Biotechnology, TX, USA), AQP4 (1:1000, Abcam Biochemicals, Grand Island, NY, USA), claudin-1 (1:1000, Invitrogen Technology, Carlsbad, CA, USA), occludin (1:1000, Invitrogen Technology, Carlsbad, CA, USA), Beclin-1 (1:1000, Cell Signaling Technology, Danvers, MA, USA), Atg5 (1:5000, Abcam Biochemicals, Grand Island, NY, USA), iFABP (1:1000, Santa Cruz Biotechnology, TX, USA), HSP70 (1:1000, Enzo Life Sciences, Inc., NY, USA), LC3 (1:1000, Sigma Aldrich) or β-actin (1:5000, Sigma Aldrich). The membranes were washed with TBST and incubated for 2 ~ 2.5 h at 25 °C with an anti-rabbit (mouse) immunoglobulin G horseradish peroxidase-conjugated secondary antibody (1:5000 dilution; ZhongShan Golden Bridge Biological Technology Co., Ltd, Beijing, China). After being washed with TBST, blots on the membrane were developed by using an enhanced chemiluminescence Western blotting kit (ECL-plus, Amersham Biosciences, Sweden), visualized and quantified in an imaging system (Alpha Innotech FluorChem FC2, CA, USA). Abundances of all proteins of interest were normalized to those for β-actin.

### Statistical analysis

All values are expressed as mean ± SEM. The incidence of diarrhea was analyzed by using *χ*^2^ analysis. The other data were performed by one-way analysis of variance. The normality and constant variance for experimental data were tested by the Levene’s test^63^. Differences among treatment groups were determined by the Duncan’s multiple range tests. All statistical analyses were performed using the SPSS 17.0 software (Chicago, IL, USA). Probability values < 0.05 were taken to indicate statistical significance.

### Ethics approval and consent to participate

The experimental procedures involving animals for the current study were carried out in accordance with the Chinese Guidelines for Animal Welfare and Experimental Protocol, and were approved by the Animal Care and Use Committee of Wuhan Polytechnic University.

### Consent for publication

All authors consent to participate and publish this article.

## Supplementary Information

Supplementary Legend.

Supplementary Figure S1.

Supplementary Figure S2.

Supplementary Figure S3.

Supplementary Figure S4.

Supplementary Figure S5.

Supplementary Figure S6.

Supplementary Figure S7.

Supplementary Figure S8.

Supplementary Figure S9.

Supplementary Figure S10.

## Abbreviations

AQP3: Aquaporin 3
β-CG: β-Conglycinin
NAC: *N*-Acetylcysteine
IL-4: Interleukin 4
NHE3: Sodium hydrogen exchanger 3
KCNJ13: Potassium voltage-gated channel subfamily J member 13
iFABP: Intestinal fatty-acid binding protein
