# Supplementary material for: N-Acetylcysteine improves intestinal function and attenuates intestinal autophagy in piglets challenged with β-conglycinin
Source: Sci Rep. 2021 Jan 13;11:1261. doi: 10.1038/s41598-021-80994-2 (PMC7807065; doi:10.1038/s41598-021-80994-2)
Supplement: Supplementary file 1 — Supplementary Legend. [file 41598_2021_80994_MOESM1_ESM.doc]

**Title:** N-acetylcysteine improves intestinal function and attenuates intestinal autophagy in piglets challenged with β-conglycinin

**Authors:** Huiyun Wang, Chengcheng Li, Meng Peng, Lei Wang, Di Zhao, Tao Wu, Dan Yi, Yongqing Hou, Guoyao Wu

**Figure legend:** Full length gels and blots for AQP3, AQP4, iFABP, claudin-1, occludin, HSP70, Beclin-1, Atg5, LC3, and beta-actin in the jejunal mucosa of piglets. Piglets in the control group (CTRL) were fed a liquid diet containing 10% casein, whereas those in the β-CG and β-CG + NAC groups were fed liquid diets containing 9.5% casein and 0.5% β-CG for 2 days. Thereafter, pigs in the β-CG + NAC group were orally administrated with 50 mg (kg BW)^-1^ NAC for 3 days, while those in the other two groups were orally administrated with the same volume of sterile saline.
