# Supplementary figures and images for: N-Acetylcysteine improves intestinal function and attenuates intestinal autophagy in piglets challenged with β-conglycinin
Source: Sci Rep. 2021 Jan 13;11:1261. doi: 10.1038/s41598-021-80994-2 (PMC7807065; doi:10.1038/s41598-021-80994-2)

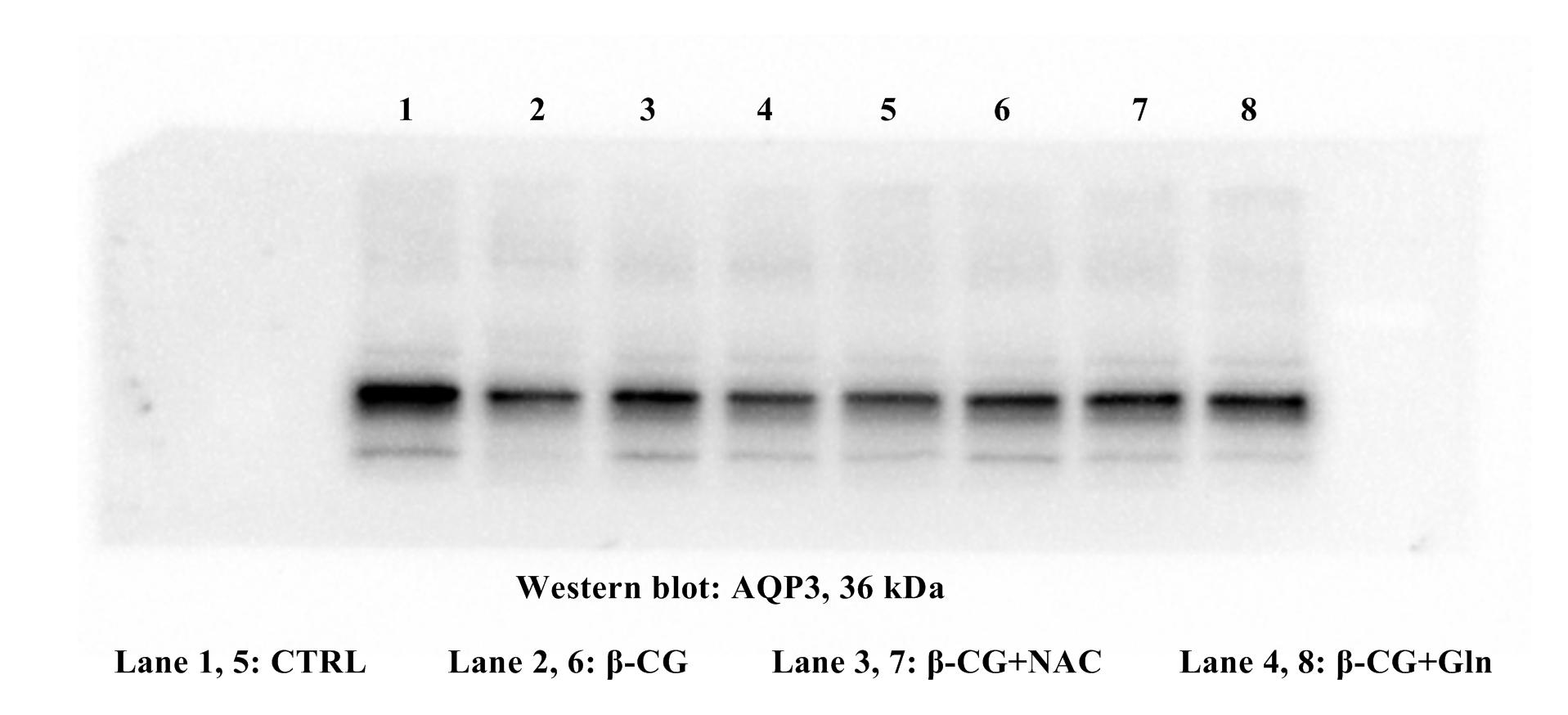

Supplement: Supplementary file 2 — Supplementary Figure S1. [file 41598_2021_80994_MOESM2_ESM.jpg]

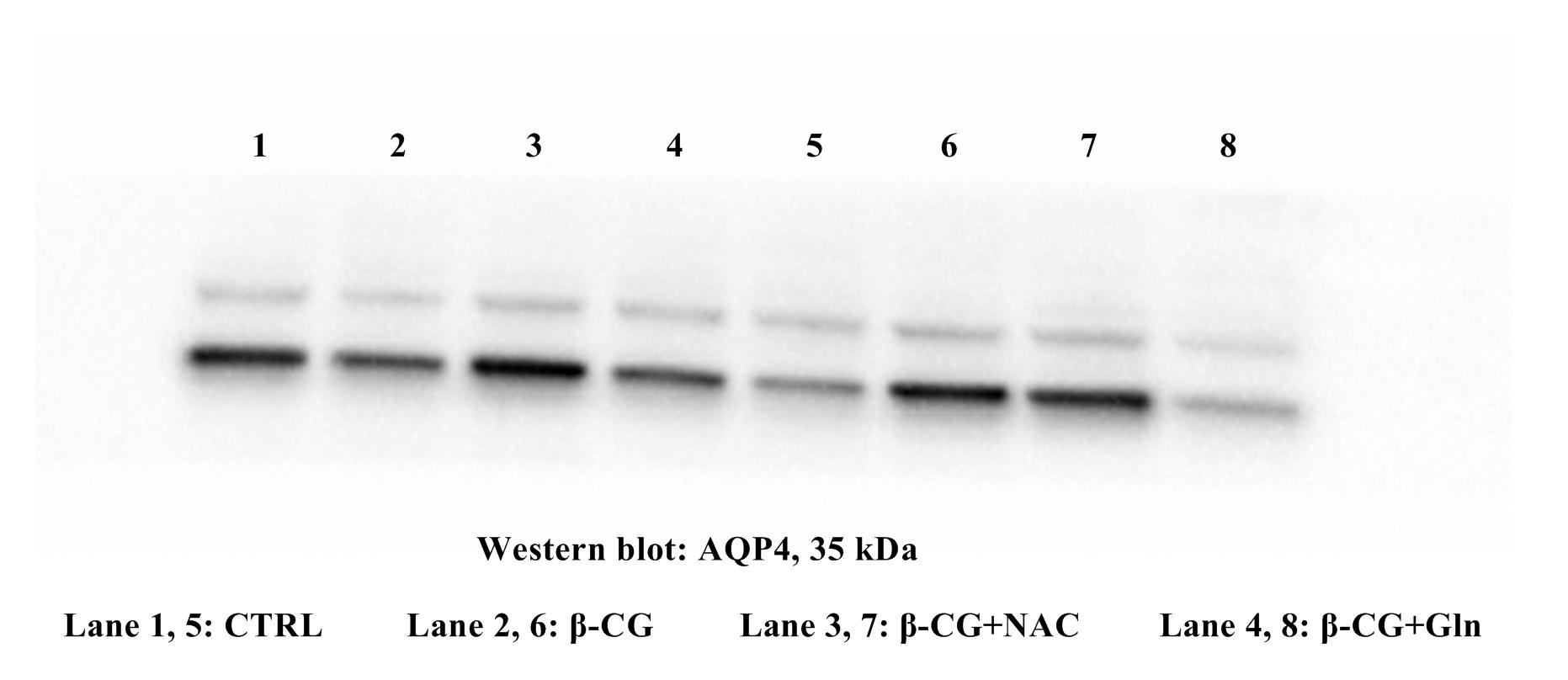

Supplement: Supplementary file 3 — Supplementary Figure S2. [file 41598_2021_80994_MOESM3_ESM.jpg]

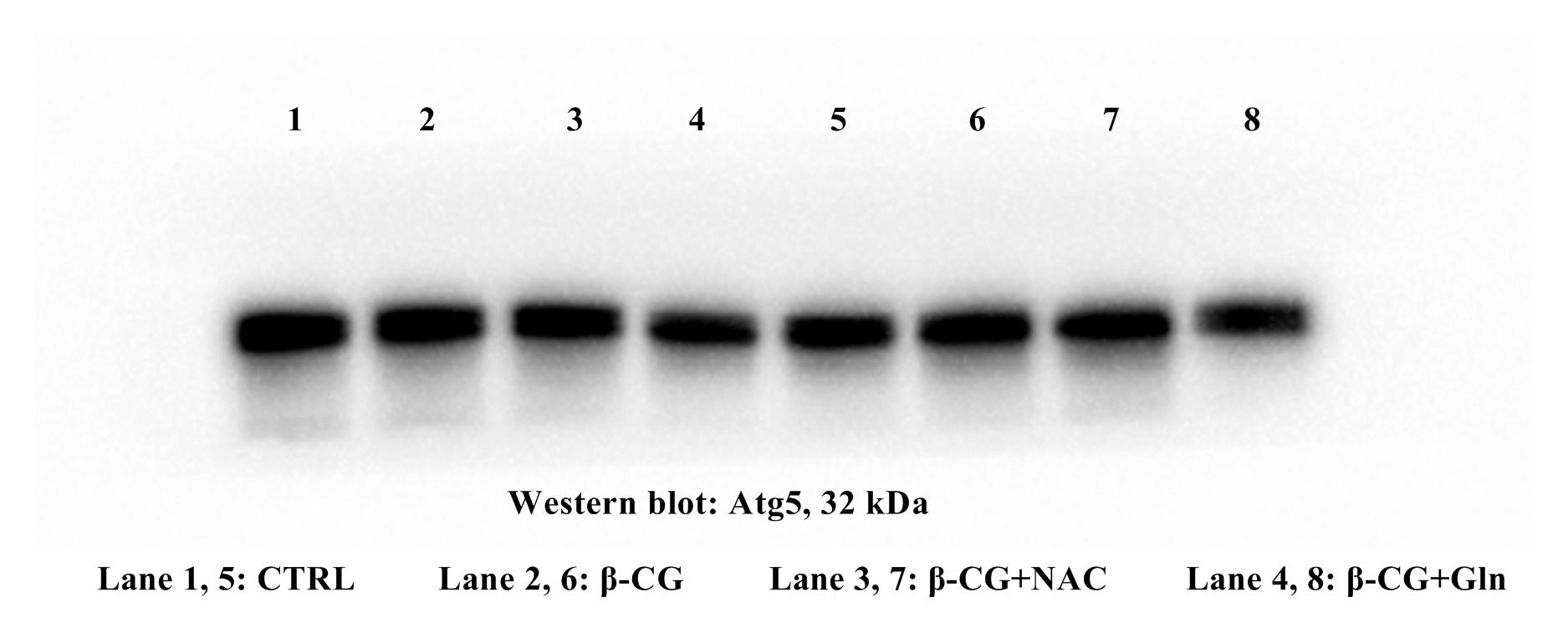

Supplement: Supplementary file 4 — Supplementary Figure S3. [file 41598_2021_80994_MOESM4_ESM.jpg]

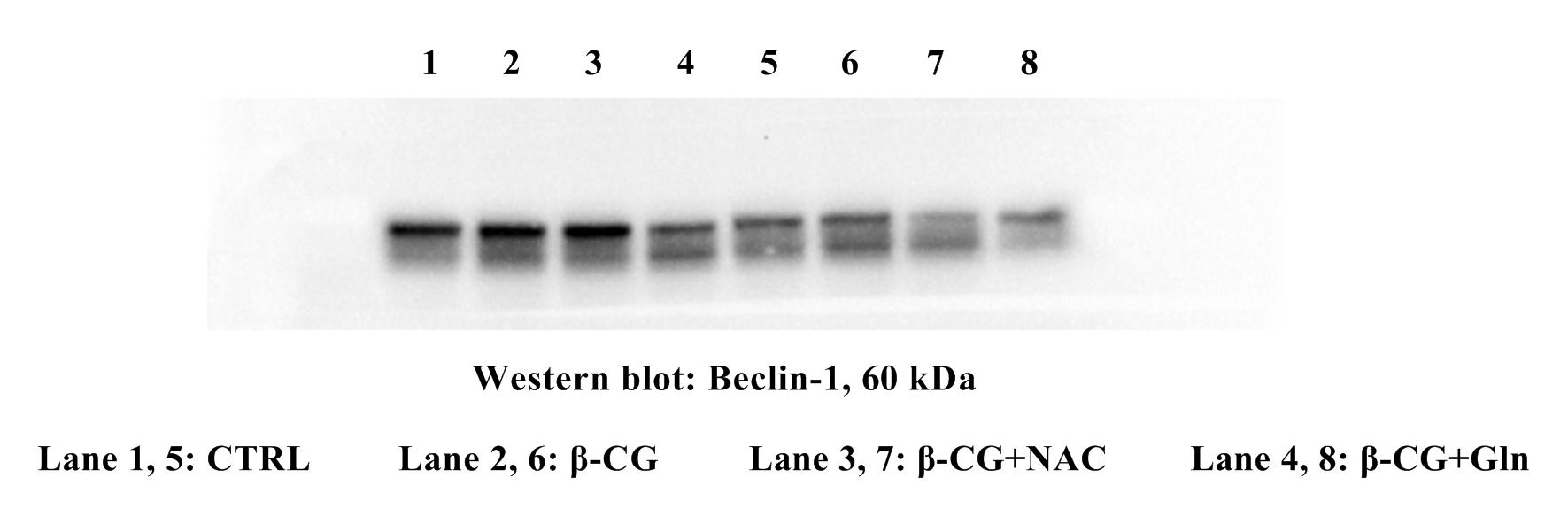

Supplement: Supplementary file 5 — Supplementary Figure S4. [file 41598_2021_80994_MOESM5_ESM.jpg]

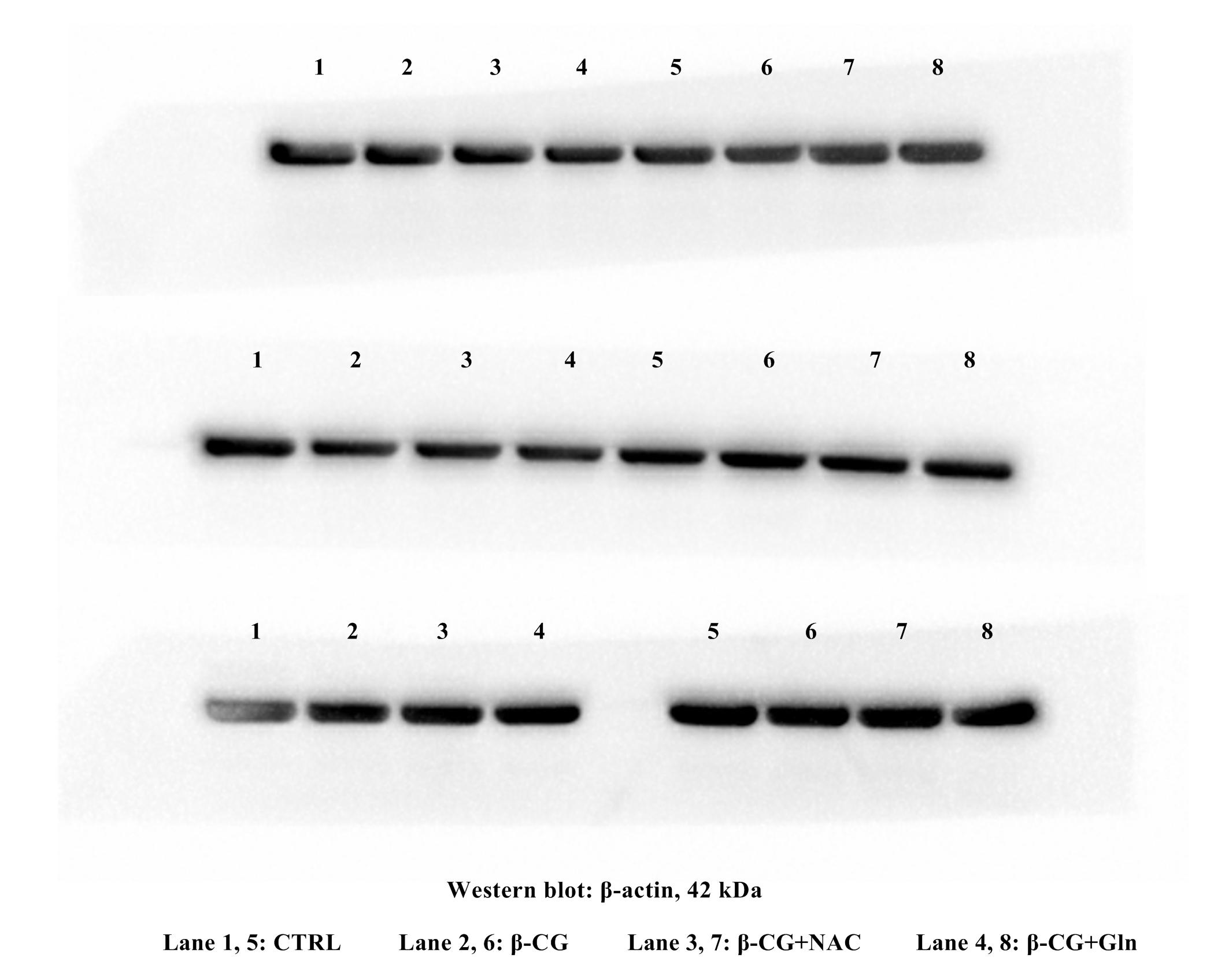

Supplement: Supplementary file 6 — Supplementary Figure S5. [file 41598_2021_80994_MOESM6_ESM.jpg]

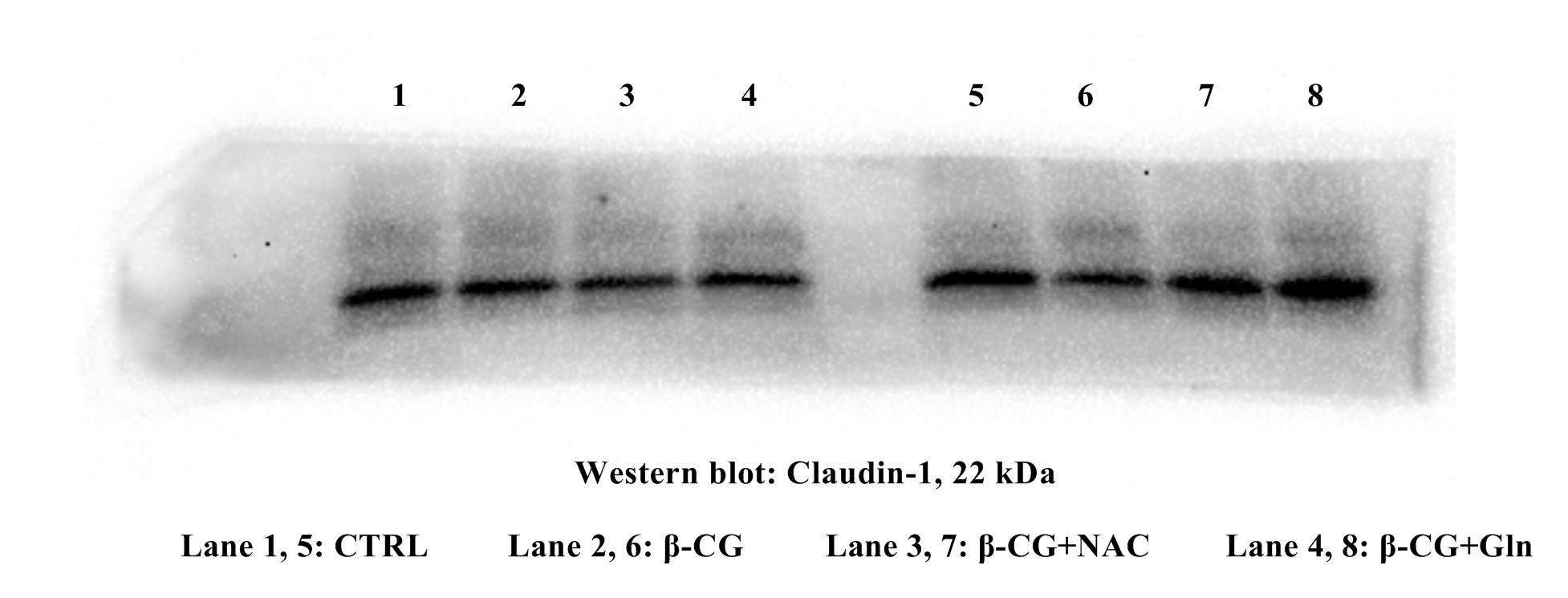

Supplement: Supplementary file 7 — Supplementary Figure S6. [file 41598_2021_80994_MOESM7_ESM.jpg]

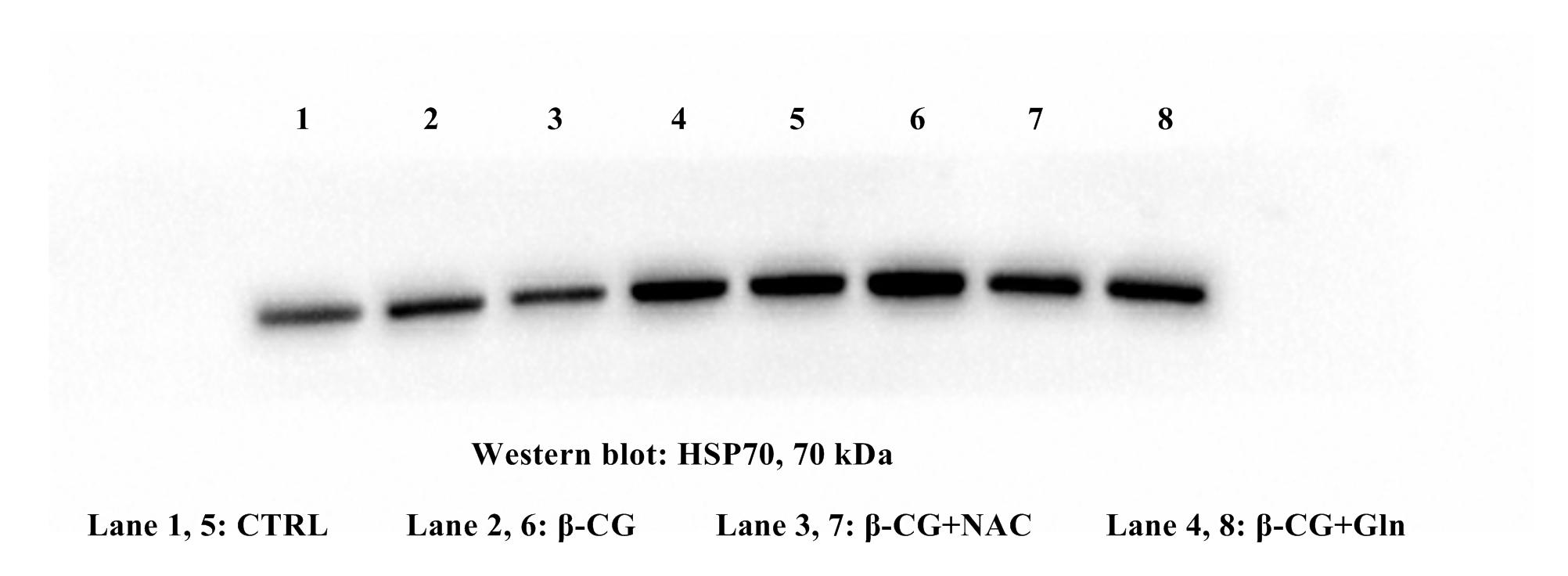

Supplement: Supplementary file 8 — Supplementary Figure S7. [file 41598_2021_80994_MOESM8_ESM.jpg]

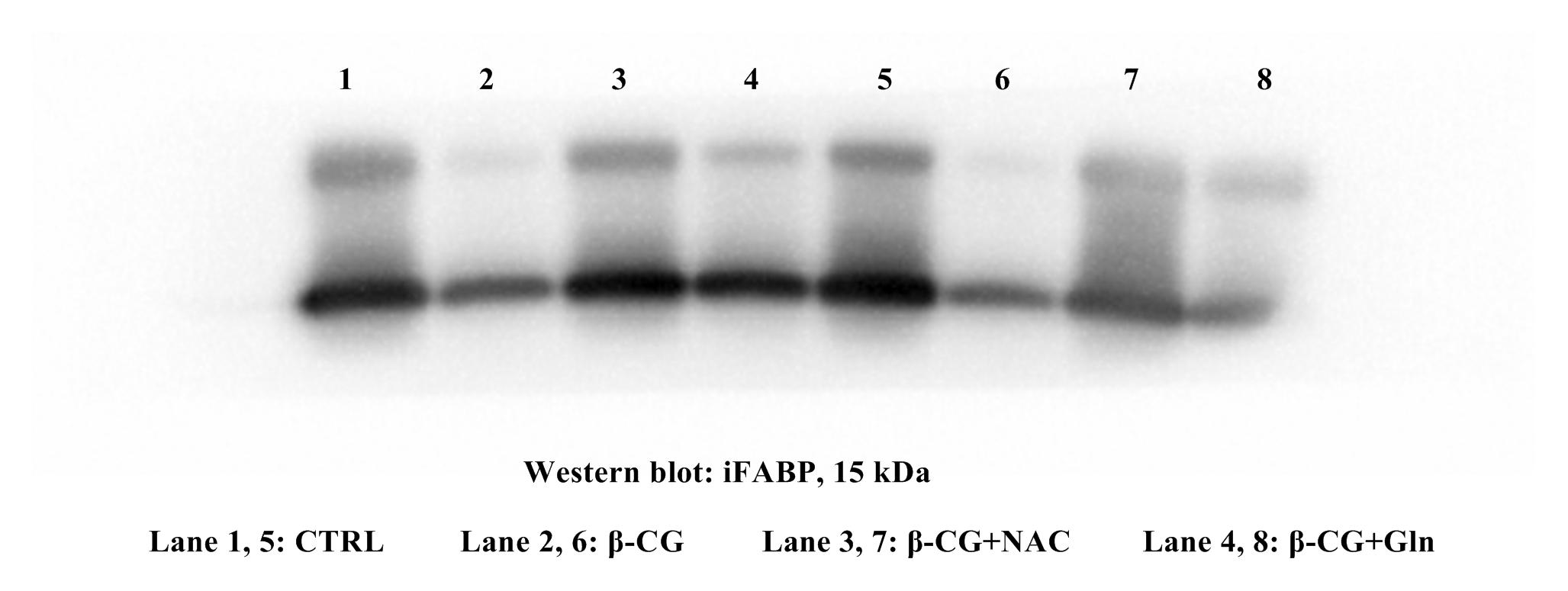

Supplement: Supplementary file 9 — Supplementary Figure S8. [file 41598_2021_80994_MOESM9_ESM.jpg]

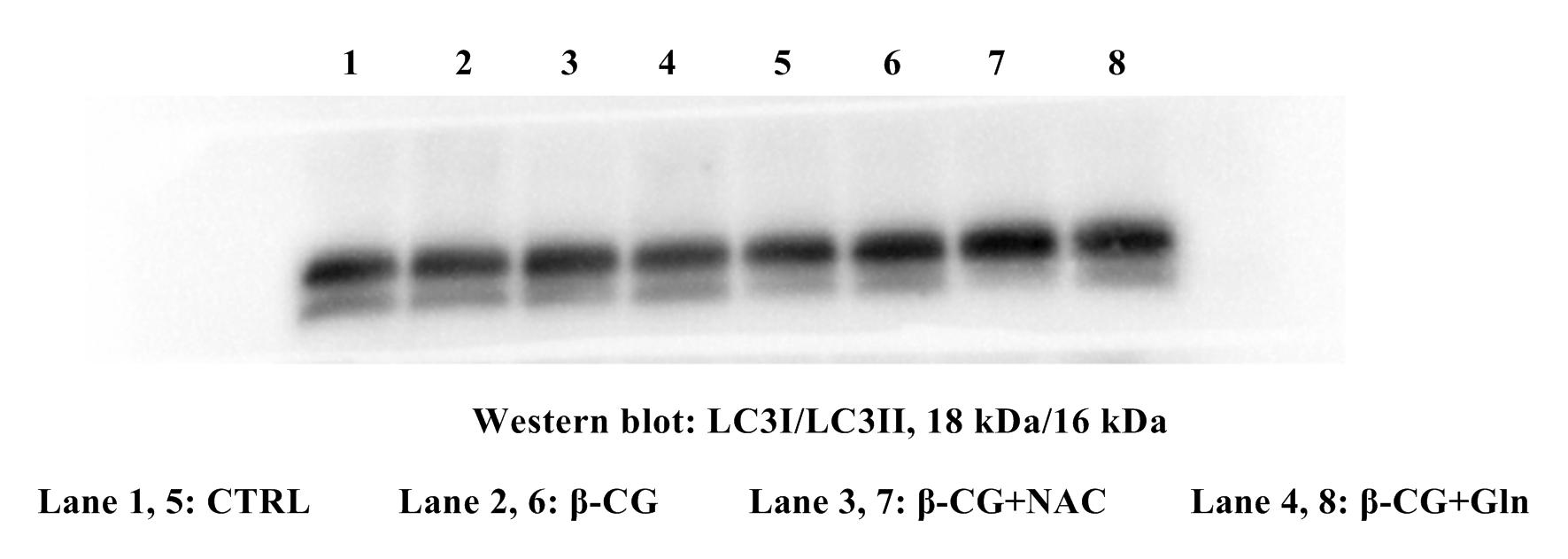

Supplement: Supplementary file 10 — Supplementary Figure S9. [file 41598_2021_80994_MOESM10_ESM.jpg]

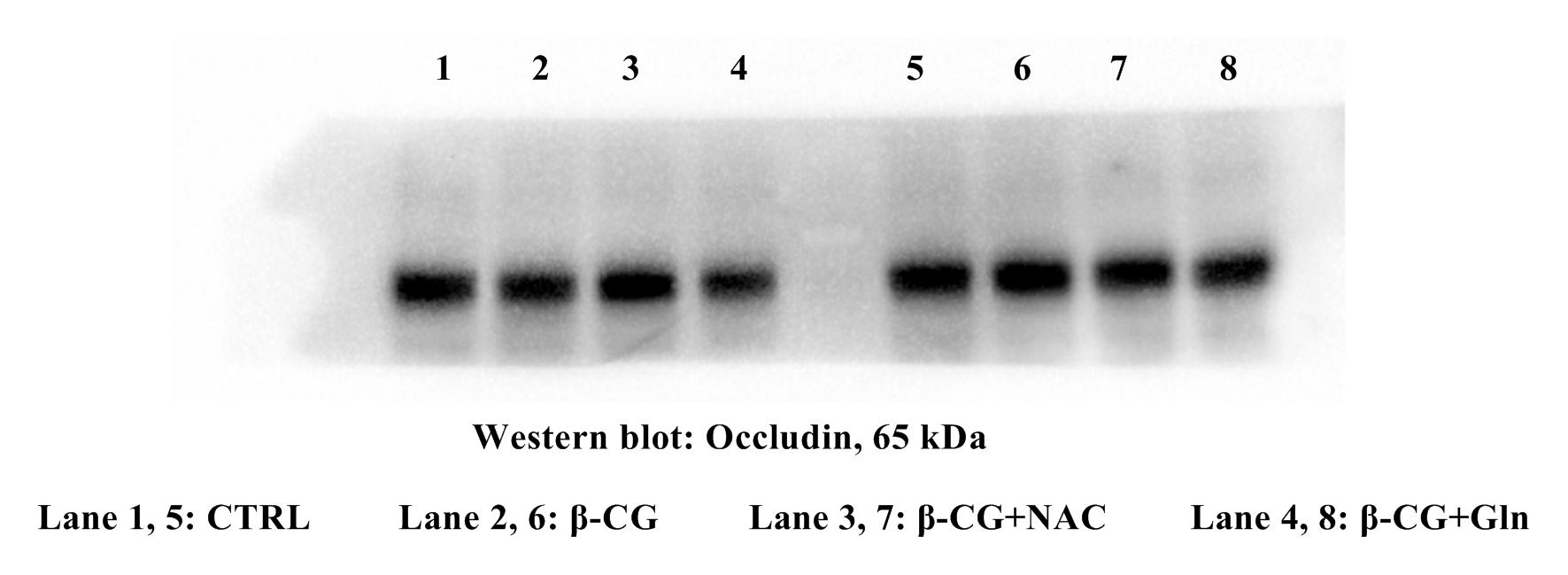

Supplement: Supplementary file 11 — Supplementary Figure S10. [file 41598_2021_80994_MOESM11_ESM.jpg]
